# Metastasis-related gene signature associates with immunity and predicts prognosis accurately in patients with osteosarcoma

**DOI:** 10.18632/aging.204902

**Published:** 2023-07-25

**Authors:** Sen Qin, Lei Li, Da Liu

**Affiliations:** 1 Department of Orthopedics, Shengjing Hospital of China Medical University, Shenyang, Liaoning, People’s Republic of China; 2 Department of Urology, Shengjing Hospital of China Medical University, Shenyang, Liaoning, People’s Republic of China

**Keywords:** metastasis-related gene, immunity, osteosarcoma, immunotherapy, drug screening

## Abstract

Osteosarcoma is the most prevalent malignant bone tumor. In this study, we identified metastasis-related genes (MRG) that are differentially expressed between primary and metastatic osteosarcoma and employed them to create metastasis-related risk tags (MRSs) for the overall survival of osteosarcoma patients. Using consistent cluster analysis, patients with osteosarcoma in the TARGET database were divided into subgroups with different metastatic scoring patterns. The clinicopathological traits, survival rates, tumor microenvironment traits, immune-related scores, and therapeutic responses of these patients varied. Additionally, we constructed MRS-based risk characteristics and nomographs and developed an MRG Score to improve patient characteristics. Thorough evaluations demonstrated that prognostic models and metastasis scores can distinguish high-risk patients from low-risk individuals, offering excellent predictive value. Finally, western blotting was used to confirm the expression of five identified MRG markers, which are crucial for osteosarcoma invasion and migration in terms of mechanism. Our findings represent a novel and practical predictive biomarker for osteosarcoma.

## INTRODUCTION

Osteosarcoma (OS) is the most common primary malignant bone cancer. It mostly affects children and teenagers, predominantly males, with an annual incidence rate of one to four cases per million people. OS is highly malignant and tends to progress rapidly [1]. OS treatments, including a combination of tumor removal and neoadjuvant chemotherapy, have been employed for decades, leading to increased prognosis and survival rates [2, 3]. Recurrent and metastatic OC, the primary cause of mortality in patients with OS, remains difficult to treat. 15 to 30% of these patients survive for five years [4, 5]. Therefore, our research aimed to identify metastasis-related genes in OS and develop reliable prognostic markers, as well as investigate the molecular mechanisms of OS metastasis. This will provide new strategies for the treatment and prognosis of OS patients.

Immunotherapy is a novel therapeutic approach that has shown promising results in the treatment of various diseases, including hepatocellular carcinoma and breast cancer [6, 7]. The tumor microenvironment (TME) is composed of various cells, such as mesenchymal cells, tumor-infiltrating immune cells (TIIC), and endothelial cells, as well as extracellular matrix molecules and inflammatory mediators. These components provide metabolites and factors that regulate various aspects of OS cell behavior, including proliferation, diffusion, dormancy, and drug resistance [8, 9]. Some researchers consider the TME to be a pivotal factor in OS development [10]. To improve the effectiveness of immunotherapy for OS, it is therefore important to conduct systematic evaluations of the immune characteristics of the TME.

The development of high-throughput next-generation sequencing and gene microarrays has made it possible to analyze gene expression patterns using bioinformatics to investigate possible causes of the disease and prospective treatment targets. Some studies have used bioinformatics to screen biomarkers in patients with OS. To identify the putative pathogenic genes for OS, Cao et al. [11] thoroughly investigated the TARGET, GSE21257, GSE39055, and GSE49003 cohorts. They then used GO and KEGG enrichment analyses to predict the functional annotations and probable pathways of the differentially expressed genes (DEG).

In this study, we screened 15 metastasis-related signals (MRS) associated with prognosis. Specifically, we investigated the interaction between genes and pharmacology by focusing on IL10RA and TLR7, which are known to suppress the production of TLR7 and have been shown to significantly enhance the migration, invasion, and adhesion of OS cells [11]. However, their bioinformatics research did not provide an in-depth analysis of the correlation between metastatic genes and the OS immune microenvironment. Metastasis-related genes (MRG) were identified by Zheng as differentially expressed genes between primary and metastatic OS and were utilized to create a novel set of six MRG prognostic markers for the overall survival of OS patients [12]. However, the authors only verified the influence of FHIT on OS metastasis and did not discuss OS treatment.

In the present study, we identified 28 metastasis-related genes (MRGs) of OS based on the target database and constructed a risk tag composed of five MRGs. Additionally, we constructed a nomogram model based on metastasis-related signatures (MRSs) to predict clinical OS. Further research was conducted to determine the connection between MRSs and immune-infiltrating cells. Investigating functional medications and potential pharmaceuticals linked to the metastatic mechanism of OS may aid in the exploration of new therapy options. This was confirmed in the OS GSE39055 and GSE21257 cohorts, and the functions of the MRSs were fully validated. We examined the effects of MRSs on the proliferation, migration, and invasion of OS cells using a functional phenotypic method and investigated the mechanism of action of MRSs in OS metastasis. Our study advances our knowledge of OS metastasis and provides new and efficient therapeutic options.

## RESULTS

### Identification of MRGs

**Figure 1:**
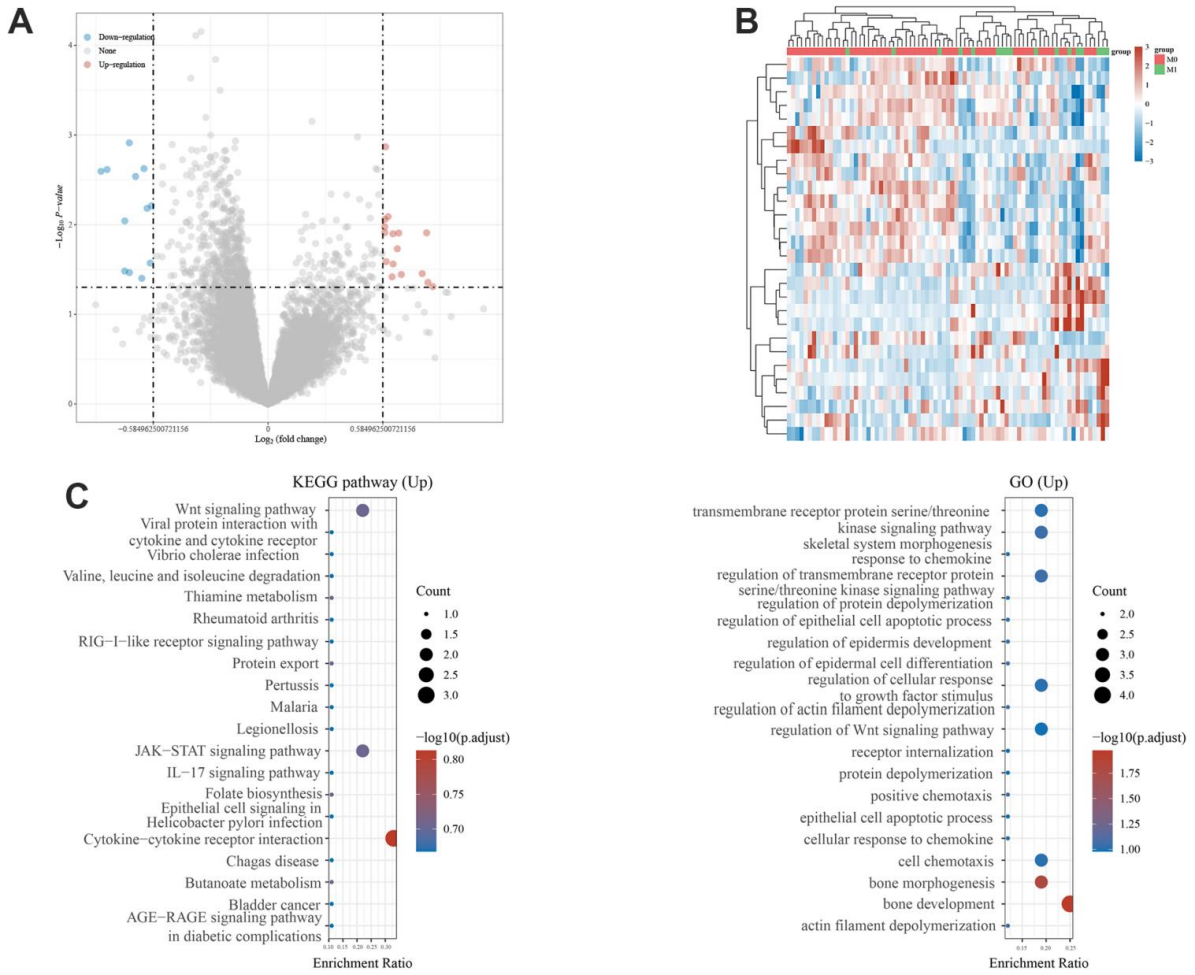
**Identification of metastasis-related genes and functional enrichment analysis.** (**A**) Volcano plot showing differentially expressed genes between primary and metastatic osteosarcoma. (**B**) Heatmaps of metastasis-related genes in the Target Database. (**C**) GO analysis of the metastasis-related genes. KEGG pathway enrichment analysis of the metastasis-related genes.

Based on metastatic and non-metastatic patients, we identified 28 DGEs. We found 16 upregulated and 12 downregulated genes (Figure 1A). The expressions of the 28 MRGs are shown in Figure 1B. Next, the upregulated genes were enriched in this pathway. GO analysis showed that it was significantly enriched in bone development, bone morphogenesis, and regulation of Wnt and kinase signaling pathways. Enrichment analysis of the KEGG pathway showed that MRGs were mainly involved in the Wnt signaling pathway, the JAK-STAT signaling pathway, and cytokine-cytotoxic receptor interactions (Figure 1C), which are known to drive the metastasis of other cancers.

### Expression pattern of OS patients based on MGRs

**Figure 2:**
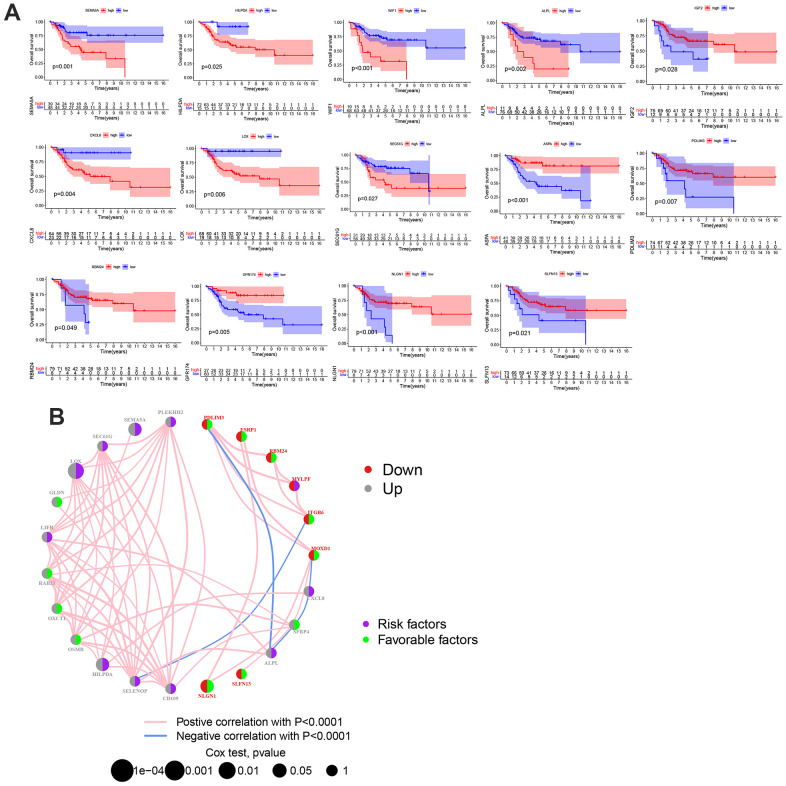
**The prognostic value of MRGs in patients with OS.** (**A**) Kaplan-Meier curves for the MRGs in OS patients from TARGET database. (**B**) A network of correlations including MRGs in the TARGET cohort. (p < 0.05 *; p <0.01 **; p < 0.001 ***).

To explore the relationship between MGRs and the prognosis of OS, we performed a survival analysis of each MGR in patients with OS from the TARGET database. High levels of SEMA5A, HILPDA, WIF1, ALPL, CXCL8, LOX, and SEC61G were significantly associated with poor overall survival in OS, whereas ASPA, RBM24, GPR174, NLGN1, SLFN13, IGF2, and PDLIM3 showed the opposite trend (Figure 2A). The interaction network showed the interactions, regulatory relationships, and risk factors between MGRs in patients with OS (Figure 2B).

### Construction of prognosis model and Norman diagram based on MGRs and identification and functional analysis of MRS

**Figure 3:**
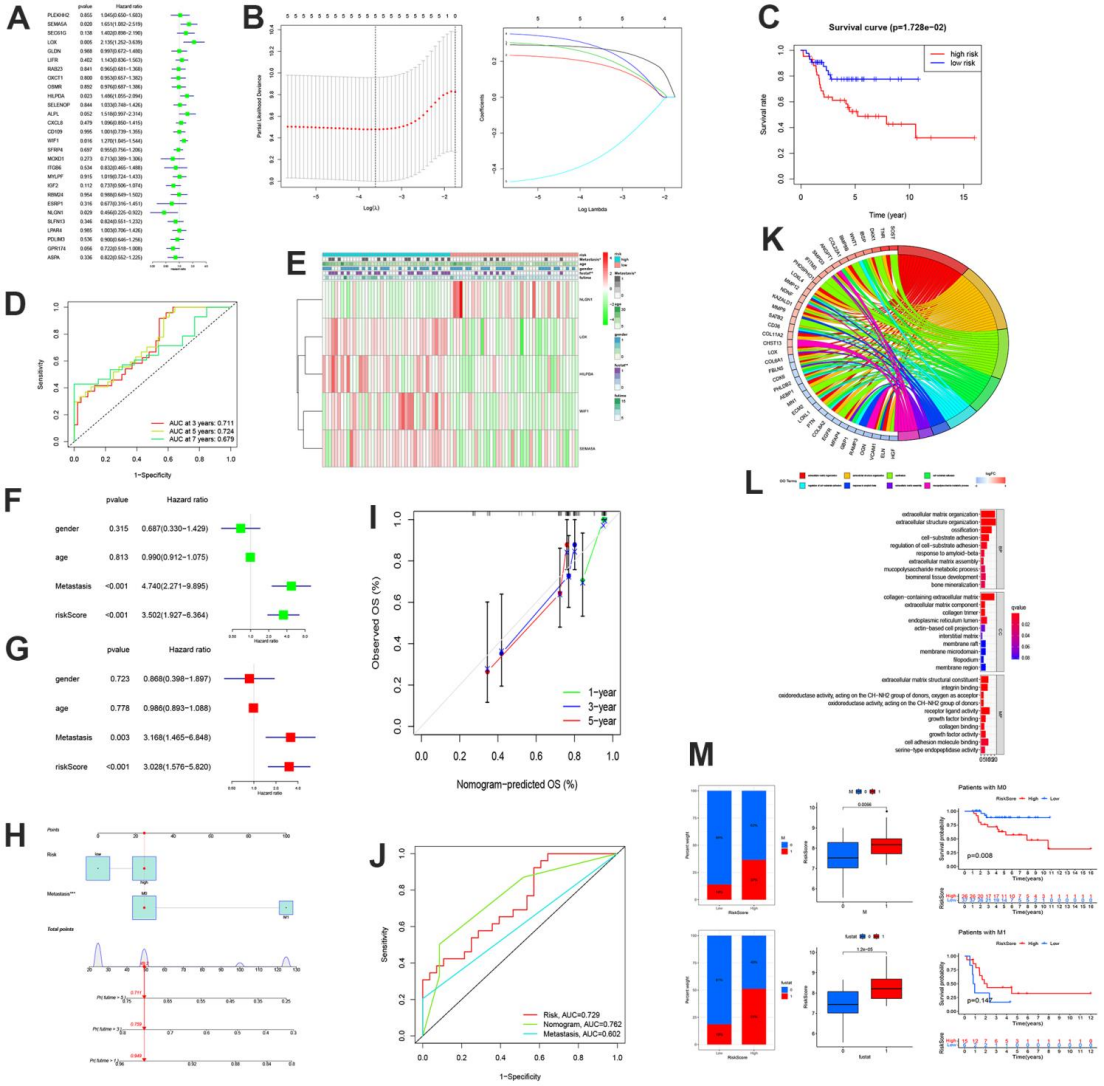
**The construction of MRSs and enrichment analysis.** (**A**) Forest plot of the univariate Cox regression analysis for MRGs. (**B**) LASSO regression and stepwise multivariate Cox regression were used to construct the five-MRG signature. (**C**) Kaplan-Meier survival analysis of patients. (**D**) ROC curve analysis of the five-MRG signature. (**E**) Expression patterns of MRSs in high-and low-risk groups. (**F**, **G**) Forrest plot of the independent prognostic factors in OS. (**H**–**J**) Nomogram for predicting the 1-, 3-, and 5-years OS of osteosarcoma patients. (**I**) Calibration curves for validating the established nomogram. (**J**) The ROC curves of the nomograms compared for 1-, 3-, and 5-years OS in osteosarcoma patients, respectively. (**K**, **L**) Circle plot through Gene Ontology (GO) analysis visualizing the biological processes enriched by DEGs. (**L**) GO analysis of differentially expressed genes between the high- and low-risk groups. (**M**) Differences in risk scores between distinct survival status and the proportion of survival status of patients.

First, based on the 28 MGRs, we conducted univariate Cox and LASSO region analyses to identify five risk genes (Figure 3A, 3B). The risk score is calculated as follows:


$$\begin{array}{l} Risk\;Score=(0.272989391508956\\ \;\;\;\;\;\;\times LOXExp)\\ \;\;\;\;\;\;+(0.18788859580782\\ \;\;\;\;\;\;\times WIF1Exp)\\ \;\;\;\;\;\;+(0.232621633568678\\ \;\;\;\;\;\;\times SEMA5AExp)\\ \;\;\;\;\;\;+(0.282143937841433\\ \;\;\;\;\;\;\times HILPDAExp)\\ \;\;\;\;\;\;+[(-0.343452464841895)\\ \;\;\;\;\;\;\times NLGN1Exp] \end{array}$$

According to Kaplan-Meier analysis, the OS of the low-risk group was considerably superior to that of the high-risk group (Figure 3C). The ROC curve also demonstrated that the model was thoroughly validated. The AUCs of the validation sets at three, five, and seven years were 0.711, 0.724, and 0.679, respectively. (Figure 3D). Additionally, a clinical correlation heat map and risk model revealed that the high-risk group had higher LOX, HILPDA, WIF1, and SEMA5A expression levels, whereas the low-risk group had higher expression levels of NLGN1.

Additionally, more individuals had metastatic disease in the high-risk group than in the low-risk group (Figure 3E). Univariate and multivariate COX analyses revealed that metastasis was a standalone prognostic risk factor for OS (P=0.01; hazard ratio [HR] =3.168) (Figure 3F, 3G). The Norman diagram includes independent prognostic criteria (Figure 3H). The calibration curve demonstrated some congruence between the observed data and the projected survival probability of the overall survival times of 1, 3, and 5 years (Figure 3I). Additionally, a comparison of the ROC curves demonstrated that the nomogram was more accurate than any clinical factor in forecasting patients’ long-term survival (Figure 3J). There were 135 MRS-related differential genes, and 39 genes were enriched in multiple pathways. These included extracellular matrix organization, regulation of cell-subtract adhesion, extracellular structure organization, response to amyloid-beta, ossification, extracellular matrix assembly, cell-subtract adhesion, and multicompolysaccharide metabolism processing (Figure 3K). In addition, BP, CC, and MF reflected the extensive heterogeneity of OS metastatic capacity (Figure 3L). We found that the group with a high-risk score corresponded to more patients with metastasis, death, and a poor prognosis (Figure 3M).

### MRS based comprehensive immunoassay and drug screening

**Figure 4:**
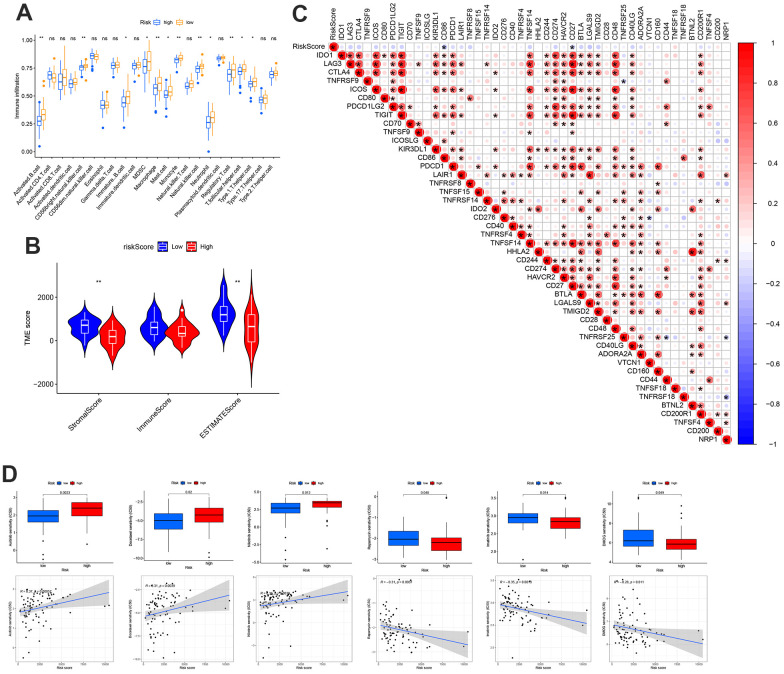
**Distinct TME characteristics and mutation of OS patients according to the risk score.** (**A**) Abundance of 23 infiltrating immune cell types. (**B**) Correlations between risk score and both immune and stromal scores. (**C**) Correlations between risk score and immune checkpoints. (**D**) Predicts the responsiveness of OS to chemotherapy.

The association between the risk score and the number of immune cells was assessed using the CIBERSORT method. The low-risk group exhibited greater immune cell infiltration than the high-risk group (Figure 4A). Additionally, the low-risk group had higher Matrix and ESTIMATE scores than the high-risk group (Figure 4B). We hypothesized that the lower risk of OS in the low-risk group may be due to greater immune cell infiltration compared to the high-risk group, which showed lower levels of immune cell infiltration. Therefore, it is necessary to explore the potential use of immune checkpoint therapies. The results of the correlation between the risk score and immune checkpoints showed that the risk score was negatively correlated with CD86 and CD27 (Figure 4C). Next, the results of drug correlation and IC50 difference showed that low-risk patients were more sensitive to Axitinib, Docetaxel, and Nilotinib, whereas high-risk groups were more sensitive to Rapamycin, Imatinib, and DMOG (Figure 4D).

### Building unsupervised consensus clustering and comprehensive immune analysis of MRGs

**Figure 5:**
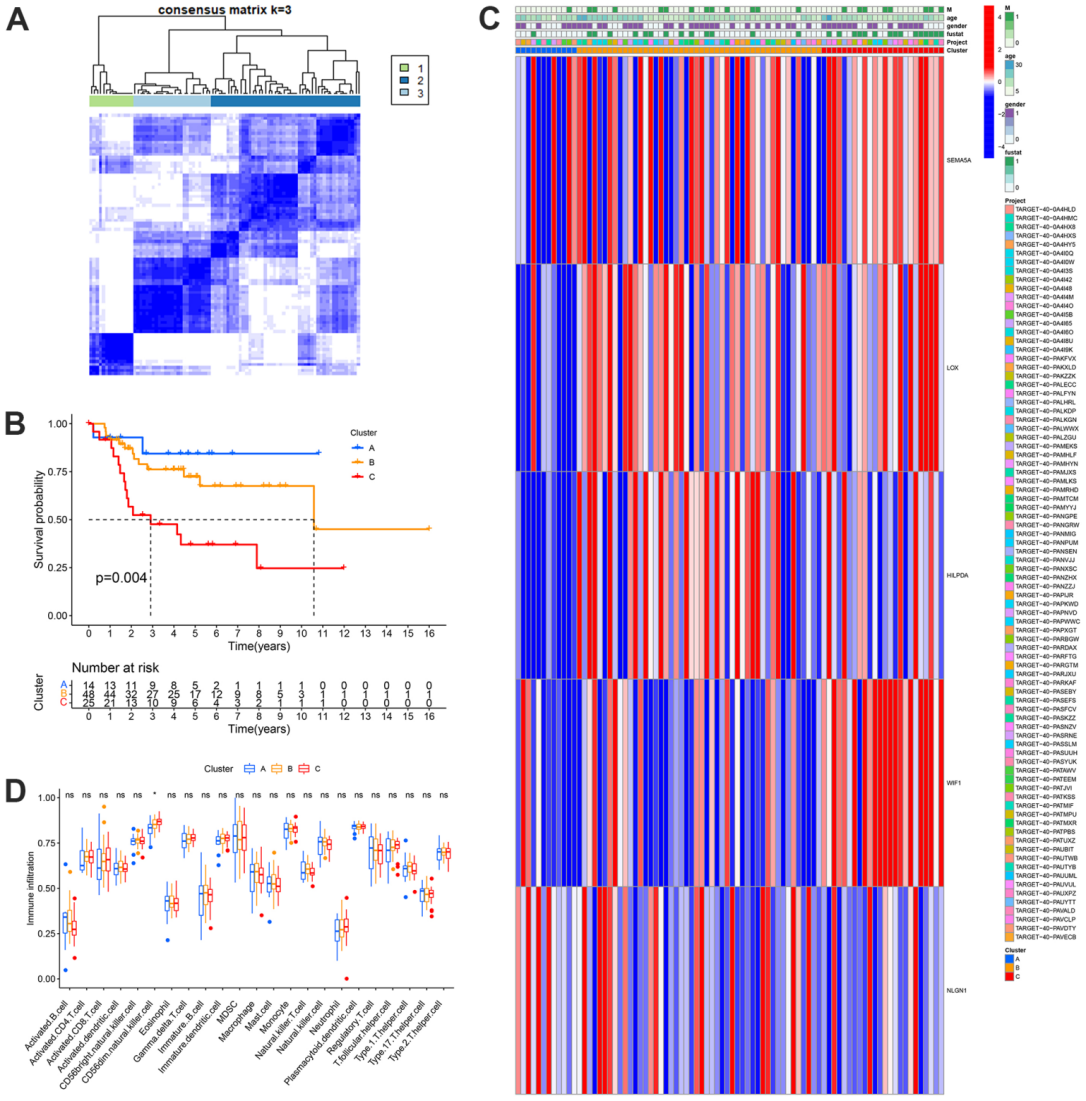
**Identification of metastasis subtypes in OS.** (**A**) t-SNE of the mRNA expression profiles of MRGs from the OS samples in the TARGET dataset confirmed the three clusters: A, B and C. (**B**) Kaplan-Meier curves for the three molecular patterns of OS patients. (**C**) Heatmap depicted the correlation between the subtypes and different clinicopathological characteristics. (**D**) Boxplots showed abundance of 23 infiltrating immune cell types.

Based on the mRNA expression levels of the 28 MGRs, OS samples in the TARGET database were clustered using unsupervised non-negative matrix decomposition. The best k value was determined using molecular clustering and comprehensive correlation analysis. We examined the k values between 2 and 9. For clustering and overall survival analysis, k = 3 was the most appropriate of all possible values (Figure 5A, 5B). Clusters A, B, and C of the MRGs were considered subgroups. Significant variations in MGRs expression and clinical traits were observed among the three groups when clinical factors and MGRs expression were compared. Among them, MRG cluster A had a good prognosis, whereas MRG cluster C had a poor prognosis. In addition, we will combine clustering with clinical features for the analysis. The findings demonstrated a substantial difference between MRG clusters A and C in OS metastasis. In addition, the expression patterns of LOX, HILPDA, WIF1, SEMA5A, and NLGN1 were consistent with the outcomes of the risk model, and the number of patients in MRG cluster A was much lower than that in MRG cluster C (Figure 5C). This is also consistent with the two survival curves that we analyzed earlier. In addition, we examined the impact of the MRG clusters on the TME and found that the infiltration of CD56dim natural killer cells increased in MRG cluster Group C (Figure 5D).

### Generation of gene cluster and comprehensive analysis of immunity

**Figure 6:**
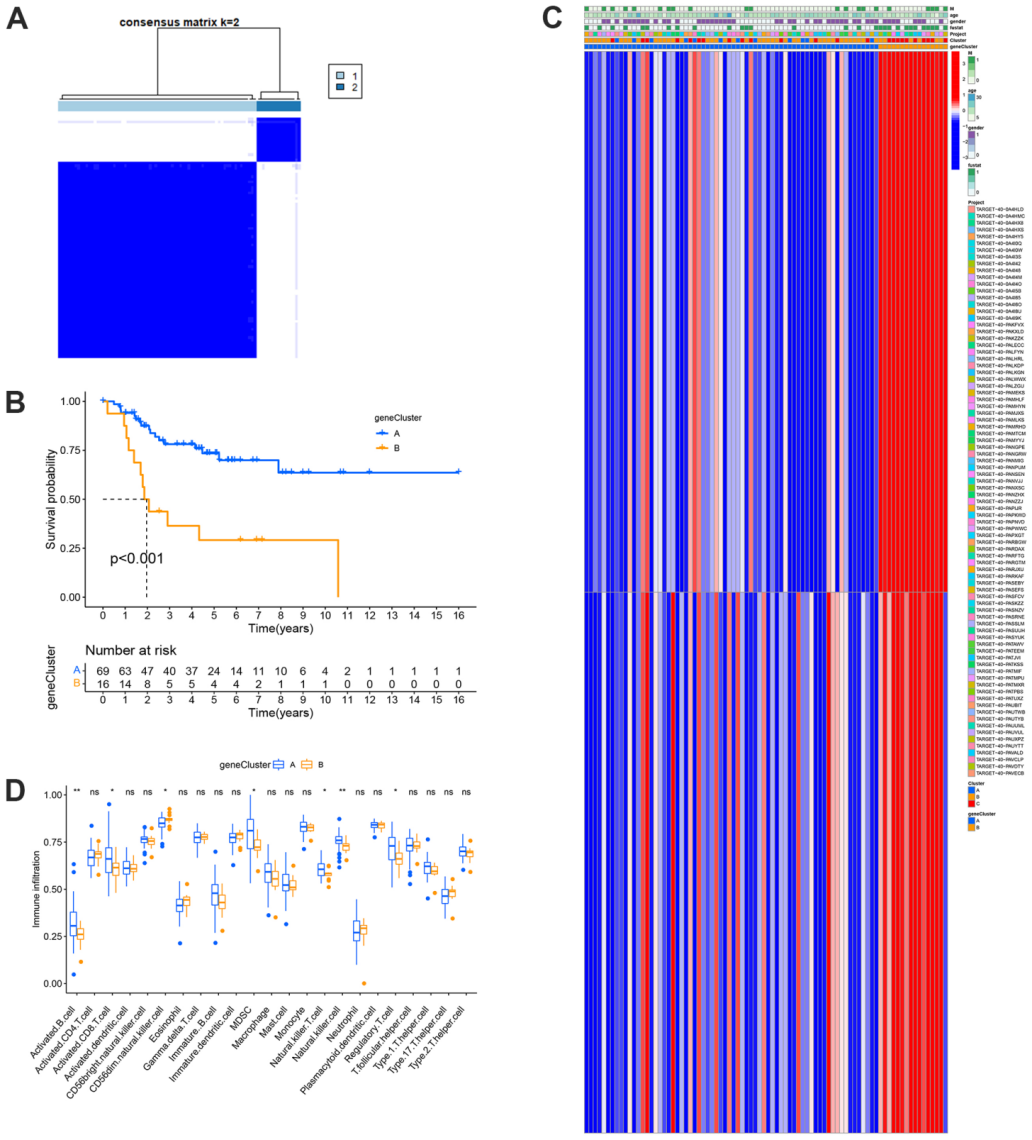
**Prognosis and TME characteristics in two metastasis gene clusters for OS patients.** (**A**) Consensus matrix heatmap defining two gene clusters according to the prognostic DEGs. (**B**) Kaplan-Meier survival analysis for patients in the two gene clusters. (**C**) Clinical features of the two gene clusters. (**D**) Boxplots showed abundance of 23 infiltrating immune cell types.

Gene clusters A and B were created based on the DEGs between the risk categories (Figure 6A). According to the survival study, patients in group B had a poorer prognosis than those in group A (Figure 6B). This previous study was consistent with the gene cluster expression pattern (Figure 6C). These genes directly affect the development and incidence of OS. Similarly, the TME characteristics of each gene cluster showed that almost all statistically significant immune cells differed significantly between the two gene clusters. Gene cluster A showed more abundant immune cell infiltration than cluster B (Figure 6D). This result shows that the TME characteristics of the gene cluster are consistent with those of the MRG clusters and the risk model.

### Construction of MRGs score and comprehensive immune analysis in patients with OS

**Figure 7:**
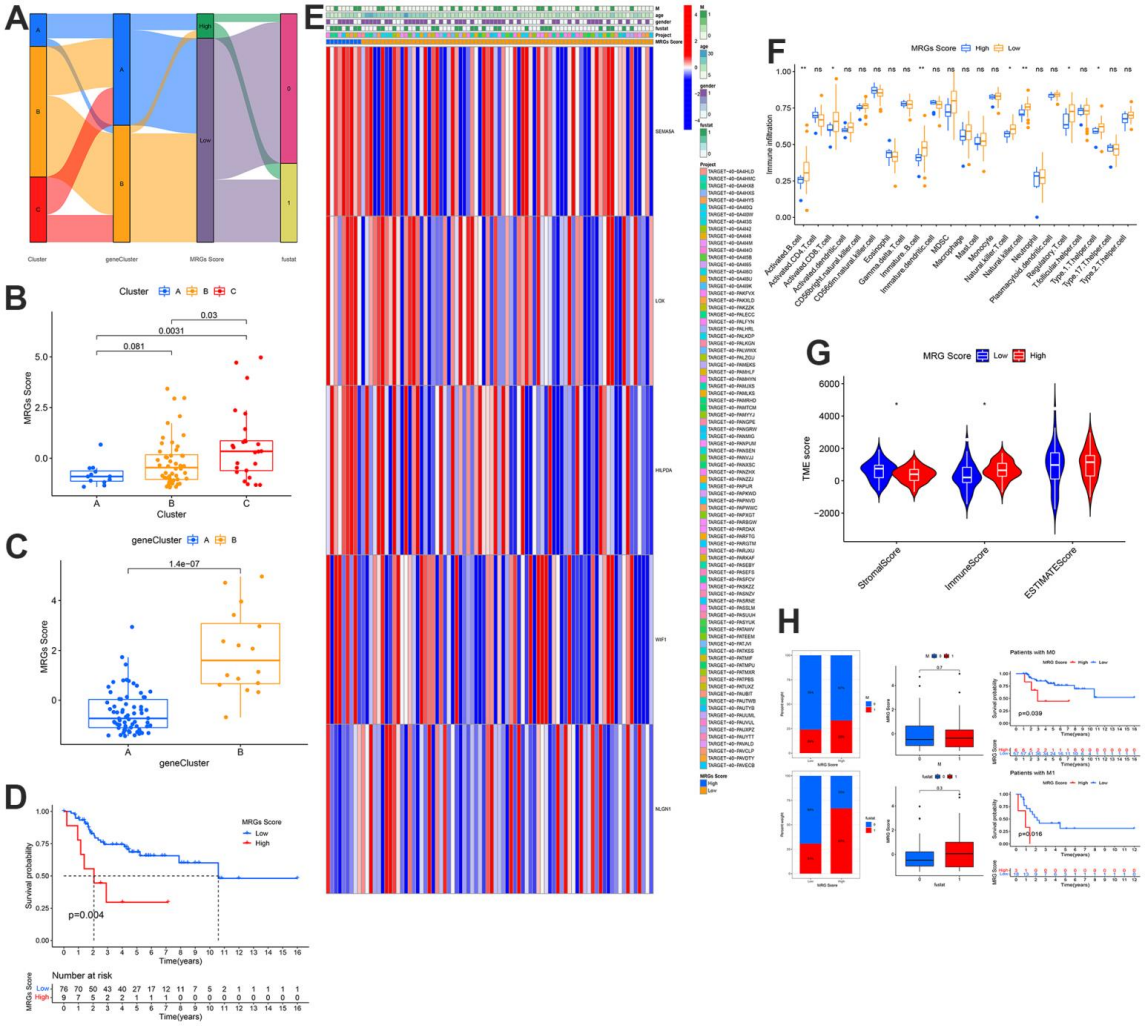
**Development and validation of the metastasis scoring system and distinct TME characteristics for OS.** (**A**) Sankey Diagram of metastasis clusters, gene clusters, metastasis score, and clinical outcomes. Differences in metastasis score between (**B**) the three metastasis subtypes and (**C**) the two gene clusters. (**D**) Kaplan-Meier analysis of the OS between the two metastasis score groups. (**E**) Heatmap depicted the correlation between the metastasis score and different clinicopathological characteristics group. (**F**) Boxplots showed abundance of 23 infiltrating immune cell types. (**G**) Correlations between risk score and both immune and stromal scores. (**H**) Differences in metastasis scores between distinct survival status and the proportion of survival status of patients.

The MRGs score and the distribution and survival of the gene and MRGs clusters are represented in the Sanggi diagram (Figure 7A). In addition, we have made further verifications. The expression of MRG scores in the gene and MRG clusters (Figure 7B, 7C). Subsequently, the survival rate was the same as before. A high MRGs score indicated a poor prognosis (Figure 7D). Interestingly, the expression patterns of LOX, HILPDA, WIF1, SEMA5A, and NLGN1 were consistent with those of previous analyses (Figure 7E). Additionally, immune cell infiltration in the low MRGs score group was more noticeable according to TME characteristic data (Figure 7F). Additionally, the matrix score of the low-risk group was higher than that of the high-risk group, and the immune score of the high-risk group was higher than that of the low-risk group (Figure 7G). The group with a high metastasis score corresponded to a greater number of patients with metastasis, death, and a poor prognosis (Figure 7H). This conclusion is consistent with that of the risk-scoring group.

### Verification of risk prediction model in external queue

**Figure 8:**
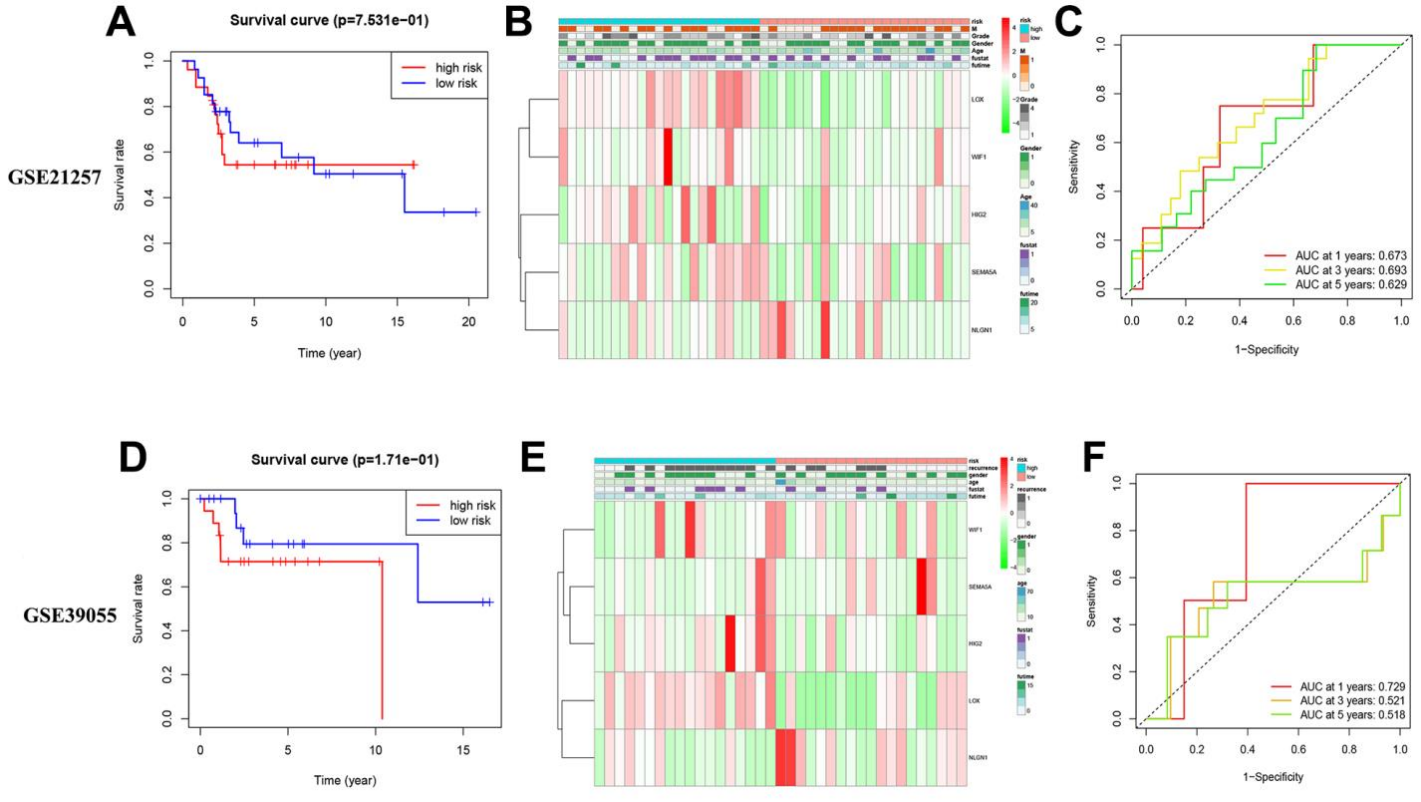
**External verification of models OS and MRSs expression pattern.** (**A**–**C**) Validation of external validation set GSE21257. (**D**–**F**) Validation of external validation set GSE39055.

We used two external queues to evaluate the reliability and stability of five MRS predictions. The risk score for each patient on these lines was determined using the same method. The patients were then divided into high- and low-risk groups according to the previously set median of the training queue. The survival rates of the two patient groups (Figure 8A, 8D) and the amount of MRS expression were mostly compatible with the expression mode of the training set risk model (Figure 8B, 8E). In the GSE21257 cohort, the area under the curve (AUC) for 1-, 3-, and 5-year survival rates were 0.673, 0.693, and 0.629, respectively. In the GSE39055 cohort, the (AUC) for 1-, 3-, and 5-year survival rates were 0.729, 0.521, and 0.518, respectively (Figure 8C, 8F).

### Differential expression of HILPDA, LOX, NLGN1, SEMA5A and WIF1 in hFOB1.19 and MG63 cells

**Figure 9:**
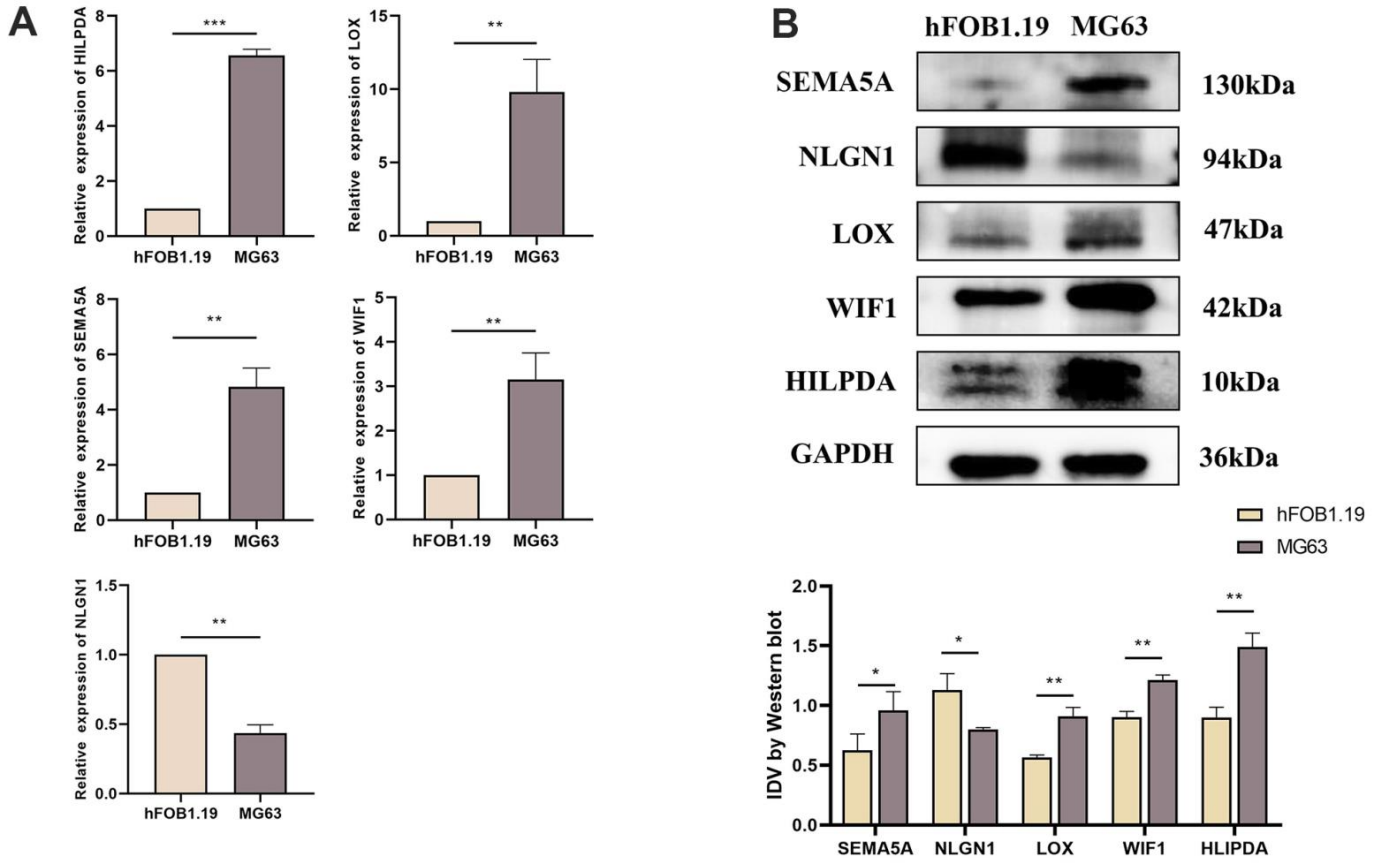
**Expression difference of HILPDA, LOX, SEMA5A, WIF1, NLGN1 in hFOB1.19 cells and MG63 cells.** (**A**) Compared with hFOB1.19 cells, the mRNA levels of HILPDA, LOX, SEMA5A and WIF1 in MG63 cells increased significantly, while NLGN1 showed the opposite. (**B**) Compared with hFOB1.19 cells, the protein levels of HILPDA, LOX, SEMA5A and WIF1 in MG63 cells increased significantly, while NLGN1 showed the opposite.(p < 0.05 *; p <0.01 **; p < 0.001 ***).

We used qRT-PCR and western blotting to study the expression of HILPDA, LOX, NLGN1, SEMA5A, and WIF1 in MG63 cells. Compared to hFOB1.19, HILPDA, LOX, SEMA5A, and WIF1 showed increased mRNA and protein expression in MG63 cells, whereas NLGN1 showed the opposite trend (Figure 9A, 9B).

### Knockdown of HILPDA, LOX, SEMA5A, WIF1 or overexpression of NLGN1 can effectively inhibit migration and invasion of MG63 cells

**Figure 10:**
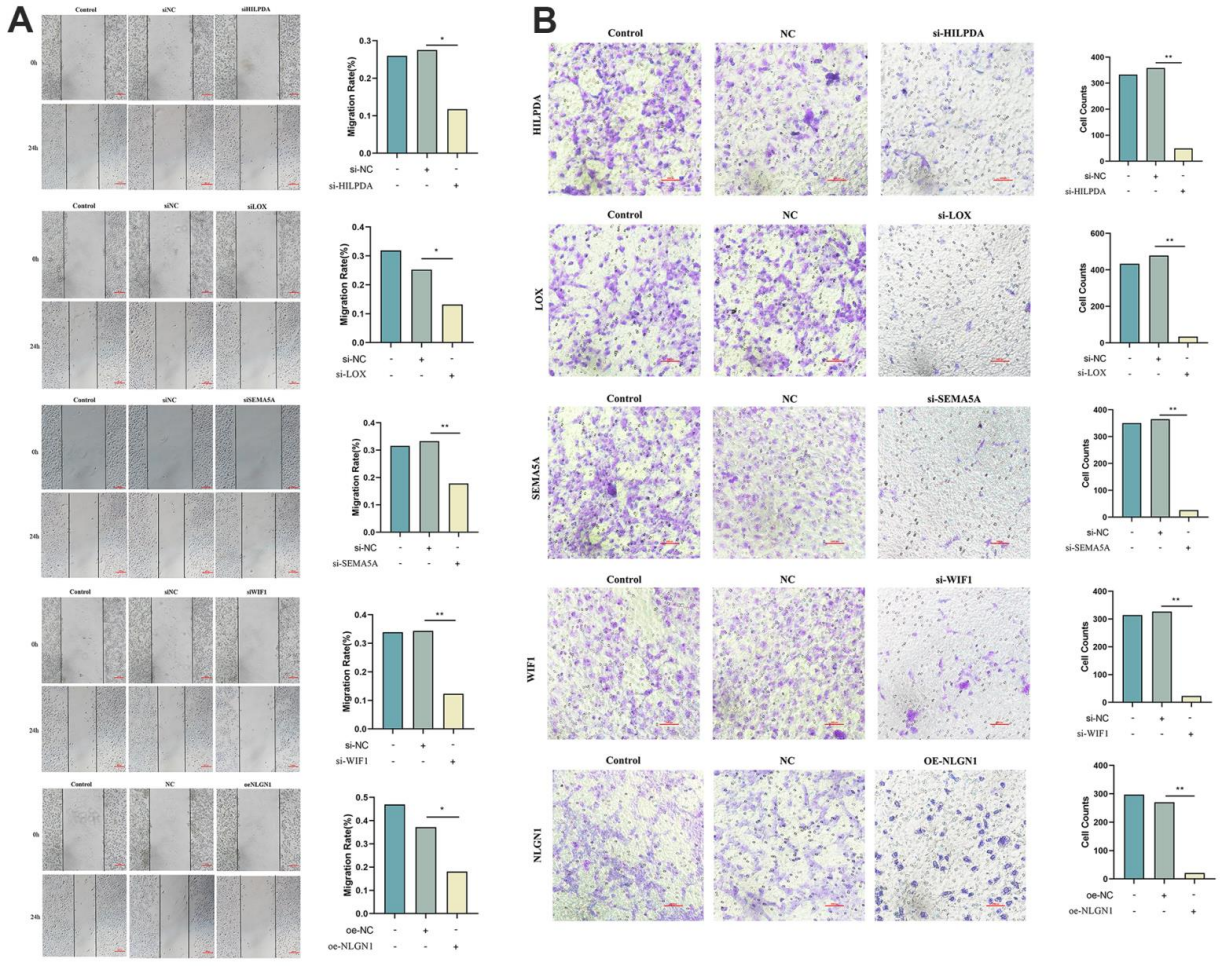
**Knock out HILPDA, LOX, SEMA5A, WIF1 or overexpress NLGN1 to observe the migration and invasion ability of MG63 cells.** (**A**, **B**) Knockdown of HILPDA, LOX, SEMA5A, WIF1 and overexpression of NLGN1 can reduce the migration and invasion ability of MG63 cells. (p < 0.05 *; p <0.01 **; p < 0.001 ***).

The scratch test was used to analyze the migration of MG63 cells. Compared to the NC group, the migration area of the si-HILPDA, si-LOX, si-SEMA5A, si-WIF1, and oe-NLGN1 groups decreased significantly, indicating that the low expression of HILPDA, LOX, SEMA5A, WIF1, and other genes and the overexpression of NLGN1 effectively inhibited the migration ability of MG63 cells (Figure 10A).

We used a Transwell assay to evaluate the invasive ability of MG63 cells. Compared to the NC group, the number of MG63 cells passing through the chamber in the si-HELPDA, si-LOX, si-SEMA5A, si-WIF1, and oe-NLGN1 groups decreased significantly, indicating that the low expression of HILPDA, LOX, SEMA5A, WIF1, and other genes and the overexpression of NLGN1 can effectively inhibit the invasiveness of MG63 cells (Figure 10B).

## DISCUSSION

OS is a bone tumor known for its high metastatic potential, with the lungs being the most common site of metastasis [13, 14]. At the time of diagnosis, 10–20% of patients will have already experienced symptoms. The incidence and development of OS tumors are complex; numerous variables control their cell growth and spread.

We used the TARGET and GEO databases to explore the characteristics of osteosarcoma (OS) and the tumor immune microenvironment (TIME), with a focus on OS metastasis and patient prognosis. Despite recent research on OS metastasis, there are currently few biomarkers available for early prediction. Therefore, it is crucial to gain a thorough understanding of the molecular process of metastasis and to discover efficient, sensitive, and specific molecular markers. For this study, we gathered information from the aforementioned databases. GO analysis revealed the wide heterogeneity of OS metastatic capacity, highlighting the need for further investigation of the molecular determinants of metastasis. To assist medical professionals in creating more successful immunotherapy regimens, we propose a metastasis score system to assess individual metastases and better understand the TME.

Firstly, we discussed the impact of 28 MRGs on the survival of osteosarcoma (OS) patients. To analyze the mRNA expression profiles of these MRGs, we employed unsupervised clustering approaches. Our findings demonstrated that the survival outcomes of OS patients varied significantly between the two groups, based on the mRNA expression profile of MRGs. Furthermore, the role of immune-related characteristics in the development and management of cancer, as well as their significance in clinical outcomes and survival, cannot be overlooked. Our comprehensive immune analysis showed remarkable consistency across various analyses, including risk models, metastasis score, gene cluster, and MRGs cluster. The findings indicated that TME infiltration was higher in the groups with good prognosis. However, research on the immune correlation of metastasis score remains limited. Hence, further studies are required to shed light on this aspect of the disease.

Secondly, we conducted corresponding experiments to validate our analysis. The results of qRT-PCR and western blot revealed that the mRNA and protein levels of HILPDA, LOX, SEMA5A, and WIF1 were increased in MG63 cells, whereas NLGN1 exhibited the opposite pattern. Moreover, the scratch test and Transwell test findings showed that overexpression of NLGN1 or knockdown of HILPDA, LOX, SEMA5A, or WIF1 effectively inhibited the migration and invasion of MG63 cells. By unraveling the process of OS proliferation and metastasis, it becomes possible to identify biomarkers for early diagnosis and assess the incidence and spread of the disease. Despite its significance, this study has some limitations that should be acknowledged. Firstly, we did not investigate the effect of metastasis score on osteosarcoma. Moreover, there is a need for further research to comprehensively understand the relationship between metastasis score and the immune microenvironment. Therefore, we urge for more in-depth studies on metastasis score in future osteosarcoma research. It is essential to identify the limitations of any study to provide a comprehensive understanding of the research and to lay the groundwork for future studies.

## MATERIALS AND METHODS

### Collection of OS datasets

The OS database was obtained from the TARGET and GEO databases. The TARGET database contained 88 tumor samples, GSE21257 contained 53 samples, and GSE39055 contained 37 samples. The TARGET database was used for the training set, and GSE21257 and GSE39055 datasets were used for the verification set.

### Screening of transfer related differential genes

The differential expression of mRNA was examined using the “limma” software package of R (version: 3.4.1), which was based on the initial count of the TARGET dataset and the associated clinical data. The screening of threshold mRNA differential expression was specified as “adjusted p = 0.05 and Log_2_ (fold change) >1 or Log_2_ (fold change) < −1“. We investigated the upregulated genes and conducted a functional enrichment analysis of the data to further validate the possible functions of the potential targets. The “ClusterProfiler” package in R was used to examine the GO function of prospective mRNA and enhance the KEGG pathway in order to better comprehend the carcinogenesis of the target gene.

### Consensus clustering analysis

Consensus unsupervised clustering analysis was carried out using the R package “CONSENSUSClusterPlus” based on the expression profiles of 28 MRGs. OS patients in the TARGET database were divided into three molecular subgroups of MRGs: clusters A, B, and C.

### Relationship between MRGs: clinical features and prognosis of OS

We evaluated the correlation among OS clustering, clinicopathological features, and survival outcomes to assess the clinical importance of various clustering approaches. The Kaplan-Meier technique was used to compare the overall survival rates.

### Construction of risk label and Norton

The weighting coefficient was determined using least absolute contraction and selection operator (LASSO) Cox regression analysis, and the risk model was created in R using the glmnet package. Based on the best critical value of the risk score, patients were separated into high- and low-risk groups, and the Kaplan-Meier technique was used to determine the survival rate of the patients. To define the ROC curve and obtain the AUC for survival analysis, use the “Survival ROC” tool in R. Finally, a Norman Chart was updated to include clinical and independent prognostic variables.

### Identification and enrichment analysis of differentially expressed genes based on risk model

To find DEGs between various risk score groups, R’s “limma” package is utilized. Genes were deemed to be expressed differently when their adjusted p-values were 0.05, and | log2 (FC) | was greater than 1.0. Finally, three DEGs were predicted to be prognostic genes and analyzed. The “clusterProfiler”, “enrichplot”, and “ggplot2” packages of R were used for the enrichment analysis of MRS-related differential genes in OS. The screening criteria were p<0.01 and q<0.05.

### Construction of MRGs score

Based on the three prognostic DGEs, we calculated the MRG scores for each patient with OS using PCA. Subsequently, the MRGs gene score was established using PCA, and principal components 1 and 2 were extracted as feature scores. The score formula was as follows: The MRGs score = Σ (PC1i+PC2i).

### TIME cell infiltration evaluation

The tumor immune estimation resource (TIMER) was utilized to evaluate the level of immune cell infiltration between different clusters using the Estimation of Stromal and Immune cells in malignant tumor tissues using Expression data (ESTIMATE) method. Single-sample gene set enrichment analysis (ssGSEA) was performed to determine the relative abundance and activity levels of each immune cell type in OS, followed by CIBERSORT analysis to assess the fraction of invading immune cells in OS patients with specific expression patterns, in order to explore differences between immune cell subtypes.

### Immune checkpoint and drug sensitivity of risk score

The immune checkpoint inhibitor response to a single sample or subtype was predicted using the Tumor Immune Dysfunction and Exclusion (TIDE) algorithm [15]. The medications sensitive to high-risk populations are screened based on the risk score and the IC50 value using the R “pRRophic” package.

### Cell transfection

Biomics Biotech created and manufactured si-HILPDA, si-LOX, si-SEMA5A, si-WIF, and their respective negative controls (NCs) (Nantong, China). Their sequences were listed in Supplementary Table 1. Genesyntech created and manufactured oe-NLGN1 (Homo sapiens neuroligin 1, transcript variant 1, mRNA; NCBI Reference Sequence: NM_001365923.2), and a negative control. Lipofectamine 3000 reagent was used to transfect MG63 cells (Invitrogen, Waltham, MA, USA) according to the manufacturer’s recommendations. The transfection efficiency is shown in Supplementary Figure 1.

### RNA extraction and qRT PCR detection

Total RNA from hFOB1.19 and MG63 cells should be extracted using TRIzol (Invitrogen), and 2ug of total RNA should be reverse-transcribed into cDNA using the TransScript One Step gDNA Removal and cDNA Synthesis SuperMix kit (TransGen Biotech, Beijing, China). The ABI Prism 7500 rapid real-time PCR equipment and TransStart Green qPCR SuperMix kit (TransGen Biotech, Beijing, China) were used to measure the expression levels of HILPDA, LOX, NLGN1, SEMA5A, and WIF1 (Applied Biosystems, StepOnePlus, Thermo Fisher Scientific, Waltham, MA USA). Actin was used as the internal reference for mRNA at the same time. Shanghai Sangong Biotechnology Co., Ltd. created all the primers (Shanghai, China). The primer sequences used in the experiments are listed in Table 1.

**Table 1:** The primer sequences of genes in this experiment.

**Target genes**	**Forward primer**	**Reverse primer**
LOX	CTGAAGGCCACAAAGCAAGT	CCAGGACTCAATCCCTGTGT
SEMA5A	GGAACCTGTGTTATAGCATGGC	GCACTGAGTCGTACCCTGG
NLGN1	GGTGCCCCATTGACTCTCTG	GTGGGTCCACATCATCCAATTTT
WIF1	TCTCCAAACACCTCAAAATGCT	GACACTCGCAGATGCGTCT
HILPDA	AAGCATGTGTTGAACCTCTACC	TGTGTTGGCTAGTTGGCTTCT
β-actin	CACCATGTACCCAGGCATTG	CCTGCTTGCTGATCCACATC

### Western blot analysis

Radioimmunoprecipitation (RIPA) lysis buffer was used to fully lyse the cells, and the resulting protein samples were collected, separated by electrophoresis, and transferred onto PVDF membrane (Millipore, Billerica, MA, USA). The PVDF membrane was then sealed with a fast-sealing solution and incubated with primary antibodies against HILPDA (diluted 1:1000; Affinity), LOX (diluted 1:1000; Proteintech), NLGN1(diluted 1:10000; Proteintech), SEMA5A (diluted 1:2000; Bioss), WIF1 (diluted 1:3000; Proteintech), and GAPDH(diluted 1:50000; Proteintech) at 4° C. The corresponding secondary antibodies were incubated at room temperature for 1.5 h. Finally, the protein bands were visualized using an ECL kit.

### Cell migration and invasion assays

The scratch test was used to analyze the migratory ability of MG63 cells. After the cells were digested, the seeds were spread onto a well plate. After the cells were digested, the seeds were spread onto a well plate. When the degree of cell fusion reached 95%, a sterile plastic tip was used to scratch the cells. After PBS washing (1-2 times), the cells were cultured in serum-free medium for 24 h. The test results were analyzed by Image J, and the migration areas of the cells in each group were compared.

The invasive ability of the MG63 cells was analyzed using a cross-pore chamber. According to 1 × 106 cells/ml, 200ul ratio was plated in a Transwell chamber (8-um; Corning, NY, USA). The same cells or 500ul MEM medium containing 10% fetal bovine serum were used in the inferior chamber and incubated for 24 h. The cells were fixed with 4% paraformaldehyde for 30 min, and 0.5% crystal violet dye was used for 20–30 min. The cells in the upper chamber were gently wiped with a cotton ball and the migrated cells were photographed under a high-magnification field of view using an inverted microscope (×200).

### Statistical analysis

All statistical calculations were carried out using R 3.4.1. One-way ANOVA was used to ascertain statistical significance in the experimental data, which are presented as the mean standard deviation. Statistical significance was set at a bilateral p value of 0.05.

### Availability of data and materials

The original contributions presented in this study are included in the article/Supplementary Material. Further inquiries can be directed at the corresponding author.

### Consent for publication

All authors have agreed on the contents of the manuscript.

## Supplementary Material

Supplementary Figure 1

Supplementary Table 1
