# Supplementary material for: Metastasis-related gene signature associates with immunity and predicts prognosis accurately in patients with osteosarcoma
Source: Aging (Albany NY). 2023 Jul 25;15(14):7219–36. doi: 10.18632/aging.204902 (PMC10415573; doi:10.18632/aging.204902)
Supplement: Supplementary Figure 1 [file aging-15-204902-s001.pdf]

SUPPLEMENTARY FIGURE

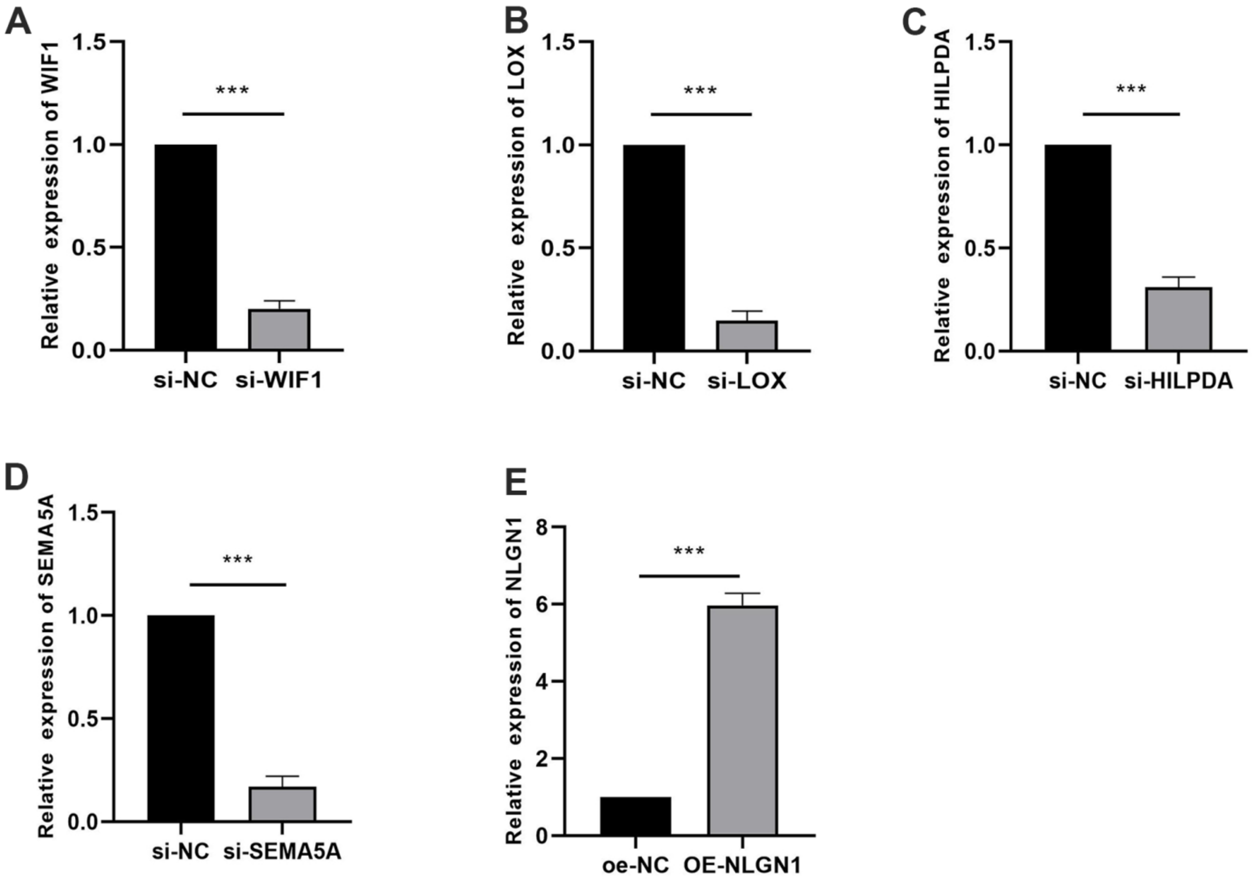

Supplementary Figure 1. The transfection efficiency confirmed by qRT-PCR. (**A**) Relative expression of WIF1 in NC or WIF1 knockdown groups. (**B**) Relative expression of LOX in NC or LOX knockdown groups. (**C**) Relative expression of HILPDA in NC or HILPDA knockdown groups. (**D**) Relative expression of SEMA5A in NC or SEMA5A knockdown groups. (**E**) Relative expression of NLGN1 in NC or overexpression groups.
