# Supplementary material for: Metastasis-related gene signature associates with immunity and predicts prognosis accurately in patients with osteosarcoma
Source: Aging (Albany NY). 2023 Jul 25;15(14):7219–36. doi: 10.18632/aging.204902 (PMC10415573; doi:10.18632/aging.204902)
Supplement: Supplementary Table 1 [file aging-15-204902-s002.pdf]

SUPPLEMENTARY TABLE

Supplementary Table 1. The siRNA sequences.

Name	Sequences
**si-LOX**	Sense: 5′-CGACAACCCTTATTACAACTA-3′<br>Antisense: 5′-GCTGTTGGGAATAATGTTGAT-3′
**si-SEMA5A**	Sense: 5′-CAAGCAGCTGTTTGCTGGAAGAGAT-3′<br>Antisense: 5′-GTTCGTCGACAAACGACCTTCTCTA-3′
**si-WIF1**	Sense: 5′-CCTCACTGTGAGAAAGCCCTTTGTA-3′<br>Antisense: 5′-GGAGTGACACTCTTTCGGGAAACAT-3′
**si-HILPDA**	Sense: 5′-ACCTCTACCTGTTAGGTGTGGTACT-3′<br>Antisense: 5′- TGGAGATGGACAATCCACACCATGA-3′
**si-NC**	Sense: 5′-TTCTCCGAACGTGTCACGTAA-3′<br>Antisense: 5′-AAGAGGCTTGCACAGTGCATT-3′
